# Therapeutic Drug Monitoring of Mycophenolic Acid Identifies Kidney Transplant Recipients Responsive to Two SARS-CoV-2 mRNA Vaccine Doses

**DOI:** 10.3389/ti.2023.11286

**Published:** 2023-06-28

**Authors:** Seraina von Moos, Elena Rho, Maria Dammann, Sanna Marjaana Kokkonen, Thomas F. Mueller, Thomas Schachtner

**Affiliations:** Division of Nephrology, University Hospital Zurich, Zurich, Switzerland

**Keywords:** kidney transplantation, therapeutic drug monitoring, seroconversion, mycophenolic acid trough level, SARS-CoV-2 vaccination

## Abstract

Immune-responsiveness to SARS-CoV-2 mRNA vaccination is reduced in kidney transplant recipients (KTRs). Previous reports point to a role of mycophenolic acid (MPA). Our observational cohort study included all KTRs at University Hospital Zurich receiving two SARS-CoV-2 mRNA vaccine doses more than 6 months post-transplantation, who were assessed by measuring anti-spike immunoglobulin G (IgG). We applied principles of therapeutic drug monitoring (TDM) to correlate MPA exposure and lymphocyte counts with SARS-CoV-2 IgG. MPA trough levels differ largely among KTRs with a median of 3.1 mg/L (range 0.7–9.5 mg/L). 34 of 84 KTRs (40%) developed positive SARS-CoV-2 IgG after two vaccine doses. KTRs who developed positive SARS-CoV-2 IgG showed significantly higher eGFR (*p* < 0.001), lower MPA trough levels (*p* < 0.001) and higher CD19^+^ lymphocytes (*p* < 0.001). MPA trough levels <2.5 mg/l and CD19^+^ lymphocytes >40/μl identify KTRs with seroconversion. Upon logistic regression, MPA trough levels <2.5 mg/L were associated with a 7-fold (CI 95%: 1.589–29.934) and ciclosporin use with a 6-fold (CI 95%: 1.148–30.853) increase in the odds of seroconversion. Our study indicates that immune-responsiveness to SARS-CoV-2 mRNA vaccines correlates with MPA exposure measured by MPA trough level but argues against a class effect of MPA. TDM-guided MPA dosing may be a strategy to increase seroconversion rate.

## Graphical Abstract

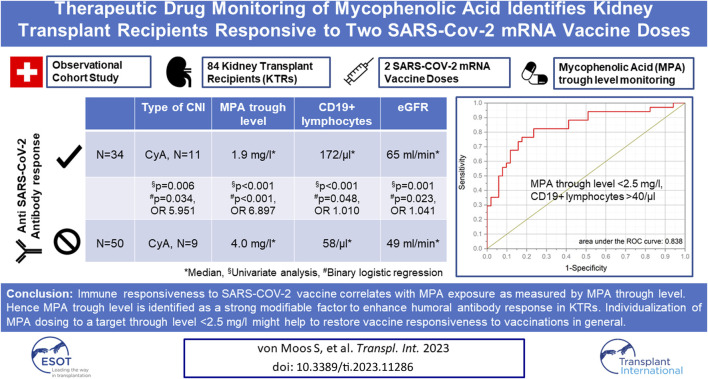

## Introduction

The appearance of severe acute respiratory syndrome coronavirus-2 (SARS-CoV-2) has changed the world. Marketing BNT162b2 (Pfizer BioNTech) and mRNA-1273 (Moderna) end of 2020 represented a milestone step toward controlling the pandemic by inducing a long-lasting protective immune response [1]. Both vaccines comprise lipid nanoparticles containing nucleoside-modified RNA encoding for the SARS-CoV-2 spike protein. Even though the exact role of cellular and humoral immune responses conferring protection is unknown, the development of neutralizing antibodies has been shown to be an immune correlate of protection against symptomatic SARS-CoV-2; [2, 3]. Yet, humoral immune responsiveness induced by mRNA-based vaccines is significantly reduced in solid organ transplant recipients, with lowest response rates in kidney and heart transplant recipients; [4–6]. Hence after two vaccinations, only 8%–40% of kidney transplant recipients (KTR) show spike-specific IgG and high frequencies of vaccine-specific T helper cells [4, 7, 8]. Also, a third vaccine dose does not substantially improve vaccine effectiveness, with only one-third of previously anti-spike IgG negative patients showing seroconversion [8–10]. A correlation of seroconversion rate with the type of vaccine, type of solid organ transplant, recipient age, years since transplantation, estimated glomerular filtration rate (eGFR), and type of maintenance immunosuppression has been shown [4, 8, 11, 12]. The strongest impairment of humoral immune responses has been reported for the use of B cell-depleting agents and glucocorticoids independent of the dose; [13]. In patients treated with B cell depleting agents, not only time since the last anti-CD20 treatment but also absolute CD19+cell counts and CD4+T-cell helper count were predictive of vaccine efficacy; [14]. Additionally, mycophenolic acid (MPA) as antimetabolite has been associated with an impaired serological response rate [4, 10–12, 15]. MPA is an inhibitor of *de novo* purine synthesis by potently inhibiting the type II isoform of inosine-5-monophosphate dehydrogenase (IMPDH), which is only expressed in activated T- and B lymphocytes [16]. Accordingly, its use has been associated with reduced frequencies of antibody-secreting plasmablasts, lower levels of IgG in the peripheral blood [17], and impeded generation of T follicular helper CD4^+^ T cells [18]. These immunological observations might explain the reduced immune responsiveness of patients treated with MPA. Based on these observations, it has been proposed by others to suspend MPA in transplant patients in the peri-vaccination period [10]. This strategy, however, can potentially increase the risk of HLA sensitization as a significant association between minimum MPA through level and formation of *de novo* DSA has previously been reported [19]. Hence, caution is recommended when completely withdrawing MPA before vaccination until this issue has been properly investigated by a randomized controlled trial.

We hypothesize that individualizing MPA dosing by applying therapeutic drug monitoring (TDM) [16, 20] might be a promising and safe strategy to improve immune responsiveness to SARS-CoV-2 vaccinations in KTRs. While MPA was initially marketed as a one-dose-suits-all drug, increasing evidence has accumulated in the past years regarding high interindividual variability of MPA exposure with a fixed dosing strategy [16, 20]. Hence, it has been shown that a fixed dosing strategy leaves a high proportion of patients outside the recommended dose range, which is important to consider in light of the narrow therapeutic window of MPA [20].

In this study, we set out to look for a correlation between MPA exposure guided by TDM and humoral immune responses as measured by spike S1 specific IgG after SARS-CoV-2 vaccination in our cohort of KTRs to find a modifiable surrogate marker with the potential to improve vaccine responsiveness to the administration of future SARS-CoV-2 vaccine doses.

## Materials and Methods

### Patients

Our study was approved by the cantonal ethic commission review board of Zurich, Switzerland (KEK-ZH-Number 2022-00013) and has been conducted in compliance with the declaration of Helsinki.

**FIGURE 1:**
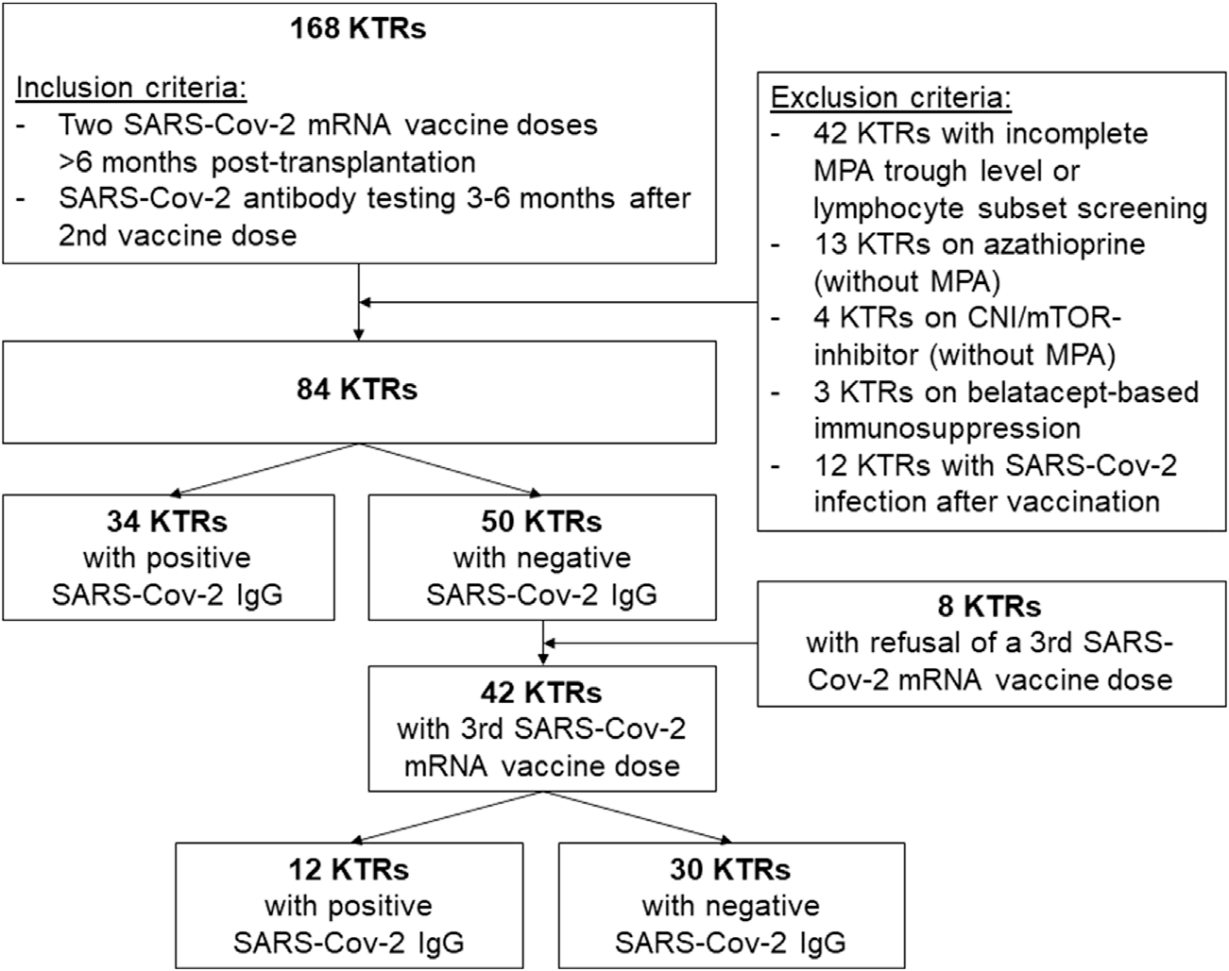
Patient inclusion and exclusion algorithm.

In this retrospective, observational cohort study, we screened 334 KTRs who underwent kidney transplantation between 1985 and 2020 and were followed at our transplant center after receiving SARS-CoV-2 vaccination. From this cohort, a total of 168 KTRs met the inclusion criteria: i) vaccination with at least two doses of either BNT162b2 (Pfizer BioNTech) or with mRNA-1273 (Moderna) against SARS-CoV-2 more than 6 months post-transplantation. This criterion ensured that none of the included patients received B- or T-cell depleting therapy within the 6 months before SARS-CoV-2 vaccination. ii) available anti-SARS-CoV-2 antibody testing between 3 and 6 months after the second vaccination. Patients who suffered from a SARS-CoV-2 infection before anti-SARS-CoV-2 antibody measurement were excluded, as well as patients with an immunosuppressive drug regimen without MPA or on a regimen with Belatacept. Additionally, patients with incomplete data regarding MPA through level measurement and lymphocyte subset screening were not considered for final analysis. Hence, a total of 84 KTRs were finally analyzed in the current study (Figure 1).

### Maintenance Immunosuppression

We selected our study for KTRs treated with an immunosuppression regimen comprising MPA together with either a calcineurin inhibitor (CNI) or a mammalian target of rapamycin (mTOR) inhibitor. Target trough levels for CNI at six and after 12 months are 80–120 ng/mL, 60–100 ng/mL and 6–8 ng/mL, 5–7 ng/mL for cyclosporine and tacrolimus, respectively. Target trough levels for mTOR (everolimus) at month six and after 12 months are 6–8 ng/mL and 4–6 ng/mL, respectively, when used in combination with MPA. MPA was either administered as mycophenolate mofetil (MMF, Cellcept^®^) or enteric-coated mycophenolate sodium (EC-MPS, Myfortic^®^). According to the immunological risk, KTRs are treated with 5 mg prednisone or steroid is withdrawn.

### Assessment of Serologic Response

The serologic response to the SARS-CoV-2 spike (S1) protein receptor-binding domain in human serum and plasma was assessed prospectively as a standard of care in our department by the commercial immunoassay Elecsys® Anti-SARS-CoV-2 S (Roche, Switzerland) as previously described [21]. This assay detects pan-Ig antibody responses and allows for a quantitative assessment of the serological response. The manufacturer states a cutoff of ≥0.8 AE/mL anti spike S1 protein to be considered as threshold for positivity.

### Assessment of Lymphocyte Subsets

Total lymphocytes, CD3^+^, CD4^+^, CD8^+^, and CD19^+^ lymphocyte counts were measured at the time of SARS-CoV-2 antibody testing. Flow cytometric determination of T and B cells was performed on a flow cytometer by Beckman Coulter (Navios Ex). The monoclonal antibodies used to identify these cell subsets were anti-CD3, anti-CD4, anti-CD8, anti-CD19, and anti-CD20, with a DuraClone IM Phenotyping BASIC Kit [22].

### Assessment of MPA Exposure

MPA trough levels were measured during SARS-CoV-2 antibody testing using high-pressure liquid chromatography (HPLC). Regarding MPA, we additionally collected data to calculate the area under the curve (AUC) using a limited sampling strategy described previously [23, 24] if appropriate data were available. The time points and formulas used for the limited sampling strategy have been shown to correlate well with the full AUC (0–12 h) [23, 24]. For MMF in combination with tacrolimus, the AUC was calculated by measuring MPA predose level (0 min) and levels 30 min and 120 min after drug intake, according to Pawinski et al. [23] For EC-MPS in combination with tacrolimus, the AUC was calculated measuring MPA predose level (0 min) as well as MPA levels 1 h, 2 h, and 4 h after drug intake and calculated according to Sanchez et al. [24].

### Statistical Methods

Statistical analysis was performed using IBM-SPSS Version 26 (SPSS, Chicago, IL, USA). Data distribution was evaluated using Shapiro–Wilk normality test and expressed as median and range. For comparisons of study groups, Mann–Whitney U-Test was used for nonparametric independent samples. Clinical characteristics were compared across groups using the Chi-square test for categorical variables. A binary logistic regression model was used to define variables associated with a positive immune response after SARS-CoV-2 vaccine doses. The Spearman’s rank correlation coefficient is used to measure the degree of association between two nonparametric continuous variables. Receiver operating characteristic (ROC) analysis was used to establish the optimal cutoff values for MPA trough levels and lymphocyte counts to identify KTRs responsive to two SARS-CoV-2 vaccine doses. Boxplots show median, interquartile range (IQR), and 95th percentile. A *p*-value of less than 0.05 is considered statistically significant.

## Results

### Overall Patient Characteristics

In total, 168 KTRs fulfilled the inclusion criteria of receiving two SARS-CoV-2 mRNA vaccine doses administered at least 6 months post-transplantation with available SARS-Cov-2 S1 IgG between three and 6 months after the second vaccine dose. After excluding KTRs with incomplete MPA or lymphocyte subset measurement, non-MPA-based immunosuppression, and SARS-CoV-2 infection before available SARS-CoV-2 antibody measurement, a total of 84 KTRs were identified for final data analysis (Figure 1). Clinical characteristics, detailed information on SARS-CoV-2 vaccination and maintenance immunosuppression, and lymphocyte counts are shown in Table 1. In total, 34 of 84 KTRs (40%) responded to two SARS-CoV-2 mRNA vaccine doses with positive SARS-CoV-2 IgG at a median period of 5 months (range 3–5 months) after the second vaccine dose.

**TABLE 1:** Clinical characteristics of 84 KTRs with positive/negative SARS-CoV-2 IgG to two doses of an mRNA SARS-Cov-2 vaccine.

	Total (*n* = 84)	Negative SARS-CoV-2 IgG (*n* = 50)	Positive SARS-CoV-2 IgG (*n* = 34)	*p-value*
Recipient characteristics				
Recipient age at transplantation, years^a^	47 (18–71)	47 (18–71)	47 (18–69)	0.834
Recipient age at 1st vaccination, years^a^	59 (19–81)	59 (19–81)	59 (33–80)	0.626
Male sex, n(%)	52 (62)	30 (60)	22 (65)	0.663
eGFR at the time of 1st vaccination, mL/min	52 (15–107)	49 (15–102)	65 (23–107)	**0.001** ^a^
Deceased donation, n(%)	63 (75)	36 (72)	27 (79)	0.441
Living donation, n(%)	21 (25)	14 (28)	7 (21)	
Simultaneous kidney/pancreas transplantation, n(%)	3 (4)	2 (4)	1 (3)	0.797
Retransplantation, n(%)	13 (15)	9 (18)	4 (12)	0.438
Primary kidney disease, n(%)				0.286
Diabetic/hypertensive	7 (8)	4 (8)	3 (9)	
Polycystic kidney disease	11 (13)	8 (16)	3 (9)	
Glomerulonephritis	34 (40)	23 (46)	11 (32)	
Others/unknown	32 (38)	15 (30)	17 (50)	
Preformed DSA, n(%)	15 (18)	11 (22)	4 (12)	0.229
*de novo* DSA, n(%)	19 (23)	12 (24)	7 (21)	0.714
SARS-Cov-2 vaccination				
Type of mRNA SARS-CoV-2 Vaccine, n(%)				0.182
BNT162b2	76 (90)	47 (94)	29 (85)	
mRNA-1273	8 (10)	3 (6)	5 (15)	
Time of 1st vaccination after transplantation, months^a^	93 (7–431)	86 (7–431)	97 (7–430)	0.179
Time of SARS-CoV-2 antibody testing after 2nd vaccination, days^a^	155 (86–196)	158 (86–196)	144 (89–192)	0.251
SARS-CoV-2 S IgG, AE/mL	-	-	21.86 (0.86–869.10)	-
Immunosuppression at the time of SARS-CoV-2 antibody testing				
Mycophenolic acid trough level, mg/L^a^	3.1 (0.7–9.5)	4.0 (1.0–9.5)	1.9 (0.7–6.8)	<0.001**
Mycophenolic acid dose per day, mg^a^				0.622
360/500^b^	4 (5)	4 (8)	0 (0)	
720/1,000^b^	19 (22)	9 (18)	10 (29)	
1,080/1,500^b^	33 (39)	20 (40)	13 (38)	
1,440/2,000^b^	28 (33)	17 (34)	11 (32)	
Calcineurin inhibitor, (%)	78 (93)	47 (94)	31 (91)	**0.016**
Cyclosporine, n	20	9	11	
Tacrolimus, n	58	38	20	
Everolimus, n (%)	6 (7)	3 (6)	3 (9)	0.682
Cyclosporine trough level, µg/L^a^	69 (30–129)	69 (32–93)	69 (30–129)	0.675
Tacrolimus trough level, µg/L^a^	5.8 (3.7–10.7)	5.9 (3.8–9.0)	5.4 (3.7–10.7)	0.422
Everolimus trough level, µg/L^a^	4.4 (4.1–5.2)	4.5 (4.2–5.2)	4.2 (4.1–4.9)	-
Prednisone, n(%)	43 (51)	28 (56)	15 (44)	0.285
Lymphocyte subsets at the time of SARS-CoV-2 antibody testing				
Total lymphocytes,/µL^a^	1,178 (327–3,450)	1,094 (327–3,450)	1,355 (709–2,828)	**0.009** ^a^
CD3^+^ lymphocytes,/µL^a^	903 (175–3,060)	834 (175–3,060)	1,004 (403–2,328)	0.056
CD4^+^ lymphocytes,/µL^a^	516 (92–1894)	500 (92–1894)	545 (256–1792)	0.132
CD8^+^ lymphocytes,/µL^a^	311 (41–4,453)	306 (41–1,159)	317 (53–4,453)	0.298
CD19^+^ lymphocytes,/µL^a^	89 (1–837)	58 (1–292)	172 (5–837)	<0.001**

^a^ Median (range).

^b^ Mycophenolate mofetil (MMF)/enteric-coated mycophenolate sodium (EC-MPS).

Significant *p* values are indicated in bold.

### Factors Associated With Positive SARS-Cov-2 IgG After Two Doses of a SARS-Cov-2 mRNA Vaccine

**FIGURE 2:**
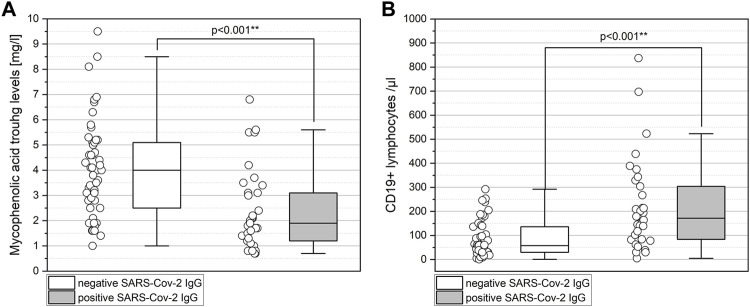
Lower MPA levels (A) and higher CD19^+^ lymphocytes (B) among KTRs who respond to two doses of the SARS-CoV-2 mRNA vaccine. **(A)** KTRs with negative SARS-CoV-2 IgG show significantly higher MPA trough levels than KTRs with positive SARS-CoV-2 IgG (*p* < 0.001). **(B)** KTRs with negative SARS-CoV-2 IgG show significantly lower CD19^+^ lymphocytes than KTRs with positive SARS-CoV-2 IgG (*p* < 0.001). Boxplots show median, interquartile range (IQR), and 95th percentile.

Upon univariate analysis, eGFR (*p* = 0.001), MPA trough level (*p* < 0.001, Figure 2A), type of calcineurin inhibitor (*p* = 0.016, Supplementary Figure S1), total lymphocytes (*p* = 0.009), and CD19^+^ lymphocytes at the time of SARS-CoV-2 antibody testing (*p* < 0.001, Figure 2B) were associated with positive SARS-Cov-2 IgG after two SARS-CoV-2 mRNA vaccine doses (Table 1).

**FIGURE 3:**
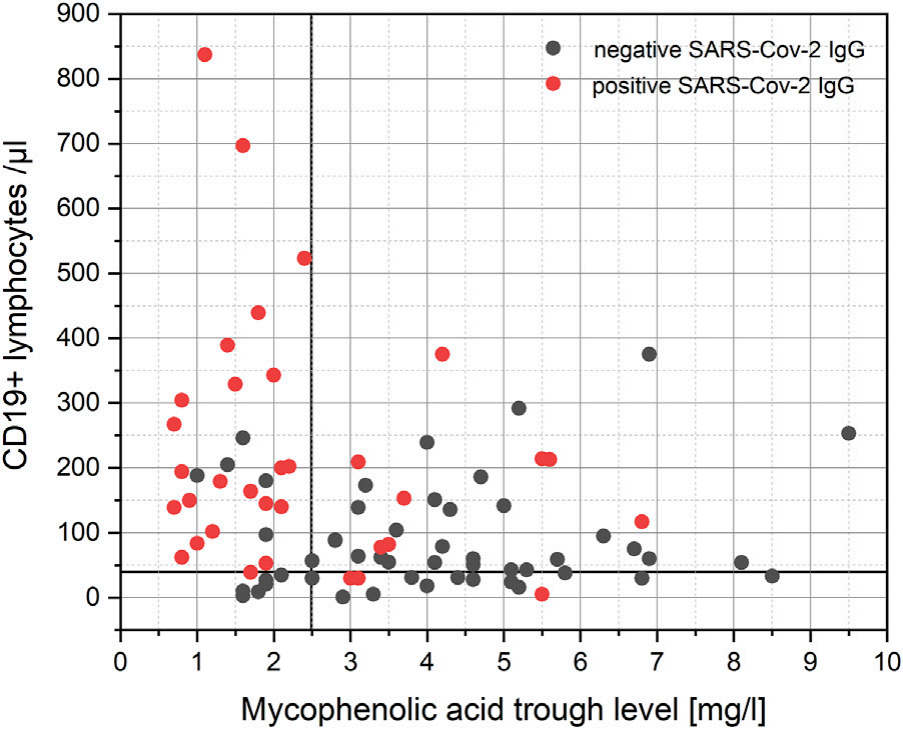
Correlation of MPA trough levels and CD19^+^ lymphocytes. MPA trough levels of less than 2.5 mg/L and CD19^+^ lymphocytes of more than 40/µL identify KTRs with positive SARS-CoV-2 IgG after two doses of SARS-CoV-2 mRNA vaccine. MPA trough levels and CD19^+^ lymphocytes show a significant negative correlation (Spearman’s correlation coefficient −0.282, *p* = 0.009).

MPA trough levels of less than 2.5 mg/L and CD19^+^ lymphocytes of more than 40/µL identify KTRs with positive SARS-CoV-2 IgG after two doses of SARS-CoV-2 mRNA vaccine with a sensitivity, specificity, positive predictive value, and negative predictive value of 67.6%, 88.0%, 82.1%, and 80.4%, respectively (Figure 3, area under the ROC curve of 0.829, *p* < 0.001)). ROC analyses are shown in Supplementary Figures S2A–C. MPA trough levels and CD19^+^ lymphocytes show a weak significant negative correlation (Spearman’s correlation coefficient −0.282, *p* = 0.009; Figure 3).

**FIGURE 4:**
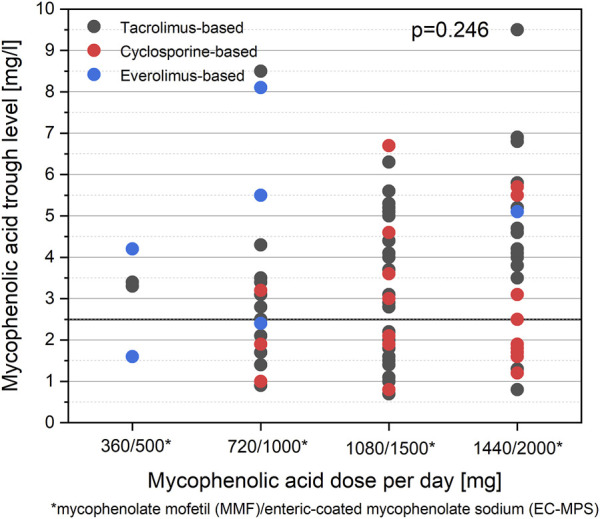
Distribution of MPA trough levels compared with MPA dosing. No differences are observed for MPA trough levels between different categories of MPA dosing per day (*p* = 0.246). No differences are observed in MPA trough levels between a tacrolimus-based and a cyclosporine-based regimen (*p* = 0.208).

Figure 4 shows the distribution of MPA trough levels compared with different MPA doses per day and accompanying immunosuppres**s**ive medication. The distribution of MPA-AUC measurements compared among a small subgroup of 14 KTRs with positive and negative SARS-CoV-2 IgG and associated MPA trough levels are shown in Supplementary Figure S3.

eGFR showed a weak significant correlation with total lymphocytes (Spearman’s correlation coefficient 0.296, *p* = 0.006), a weak significant correlation with CD3^+^ lymphocytes (Spearman’s correlation coefficient 0.224, *p* = 0.041), and a moderate significant correlation with CD19^+^ lymphocytes (Spearman’s correlation coefficient 0.489, *p* < 0.001; Supplementary Figures S4A–E).

Upon binary logistic regression, MPA trough levels <2.5 mg/L were associated with a 6.897-fold increase in the odds of seroconversion (CI 95%: 1.589–29.934; *p* = 0.010) and ciclosporin as the calcineurin-inhibitor type was associated with a 5.951-fold increase in the odds of seroconversion (CI 95%: 1.148–30.853). Of note, no collinearity was observed between MPA through level and ciclosporine use indicating independent effects (Supplementary Table S1). eGFR at time of vaccination was associated with a 1.041-fold increase in the odds of seroconversion (CI 95%: 1.005–1.078; *p* = 0.023), and CD19^+^ lymphocyte counts with a 1.010-fold increase in the odds for seroconversion (CI 95%: 1.000–1.021; *p* = 0.048, Table 2).

**TABLE 2:** Binary logistic regression model representing factors associated with the development of SARS-CoV-2 IgG after two doses of an mRNA SARS-CoV-2 vaccine.

	β	OR	CI 95%	*p-value*
eGFR at the time of 1st vaccination, mL/min/1.73 m^2^	0.040	1.041	1.005–1.078	0.023*
Mycophenolic acid trough level <2.5 mg/L	1.931	6.897	1.589–29.934	0.010*
Total lymphocytes,/µL	0.000	1.000	0.998–1.003	0.763
CD3^+^ lymphocytes,/µL	−0.002	0.998	0.994–1.002	0.398
CD19^+^ lymphocytes,/µL	0.010	1.010	1.000–1.021	0.048*
Ciclosporin as calcineurin inhibitor	1.784	5.951	1.148–30.853	0.034*

## Discussion

Even though publications reporting on reduced immune responses after SARS-CoV-2 mRNA vaccination in KTRs are accumulating [4–11, 13, 25], measures for individualized vaccination responsiveness are urgently needed. Despite clear evidence that up to one-third of patients still do not develop seroconversion even after three vaccine doses [8], no clear recommendations have emerged on improving immune responsiveness in this vulnerable cohort, even though evidence for a strong effect of antimetabolite treatment is growing [4, 10, 11, 15, 26]. Our study goes beyond the correlation of MPA dose and vaccine responses but measures MPA through levels, thereby discovering a highly promising and modifiable biomarker predictive of immune responsiveness. We analyzed the humoral immune responses after SARS-CoV-2 mRNA vaccinations in a total of 84 KTRs. In this cohort, 40% (34/84) showed seroconversion after two doses of the SARS-CoV-2 mRNA vaccine, which is in line with the literature [8]. There was no difference between the two groups concerning classically reported risk factors for reduced immune responsiveness, such as recipient age at vaccination or time since transplantation. With respect to immunosuppression, calcineurin inhibitor trough levels were similar between the two groups, as was the use of prednisone. However, upon multivariate analysis, MPA trough levels were significantly lower in KTRs with positive anti-spike IgG response after two vaccine doses and use of ciclosporine as calcineurin inhibitor type was more frequent in responders. Importantly and in contrast to a recently reported study by Kantauskaite et al.; [11], we did not observe a difference in cumulative MPA dose between the groups. This fact underscores the high interindividual variability of MPA exposure with a fixed dosing strategy based on the complex pharmacodynamic and pharmacokinetics of MPA; [16, 20]. Especially when combined with tacrolimus, drug concentrations in the toxic range are more commonly encountered than in combination with ciclosporin, which inhibits enterohepatic cycling (EHC) [16, 20].

So far, trials investigating the benefit of TDM for MPA primarily focused on the relationship between low MPA exposure as measured by AUC and under-immunosuppression reflected by rejection. Here, a clear correlation between MPA-AUC <30mgxh/L and rejection rates could be observed with the recommendation to target an MPA-AUC of 40mgxh/L [27, 28]. Until now, less attention has been devoted to MPA overexposure [27]. Nevertheless, according to the literature, an MPA-AUC above 60 mgxh/L has been suggested to be associated with adverse events [20]. While MPA-AUC measurement is the gold standard when applying TDM for MPA(16, 20); such practice is cumbersome during daily routine requiring at least three blood samples taken at different time points. Measurement of MPA trough level is much more convenient yet highly debated. Nevertheless, MPA-AUC and MPA trough levels have been shown to correlate with an MPA trough level of approximately 1.4 mg/L, corresponding to an AUC >30 mgxh/L [29]. The OPTICEPT trial, which also showed correlations between MPA-AUC and through levels, targeted MPA throughs of ≥1.3 μg/mL or ≥1.9 μg/mL for ciclosporine and tacrolimus treated patients, respectively [30]. In the present study, we found an MPA trough level of less than 2.5 mg/L being associated with a positive humoral immune response to SARS-CoV-2 mRNA vaccination. In a small subset of KTRs, we could confirm that an MPA trough level of <2.5 mg/L corresponds to MPA-AUC <60 mgxl/h. Hence, higher levels, especially trough levels above 4 mg/L, are likely to correspond to MPA overdosage, reflecting an MPA-AUC >60 mgxh/L. Our data indicate that TDM measuring MPA trough levels is promising for identifying KTRs that respond to SARS-CoV-2 mRNA vaccination. The previously reported observation of reduced vaccine responsiveness in KTRs treated with MPA might therefore not be a class effect but rather a matter of dosing, urgently calling for TDM for this drug with a narrow therapeutic window. We hypothesize that immune responsiveness in KTRs treated with MPA can be restored by individually adapting MPA dosage to a target range.

In addition to MPA trough level <2.5 mg/L, our analysis suggests better responsiveness to two SARS-CoV-2 vaccine doses in KTRs on a ciclosporin-based compared to a tacrolimus-based immunosuppressive regimen independent from MPA trough levels and the well-known impact of ciclosporin on MPA pharmacokinetics. Several independent studies found *in vitro* evidence of ciclosporin-mediated inhibition of SARS-CoV-2 replication, which led to speculation that ciclosporin could be used as the preferred calcineurin inhibitor during SARS-CoV-2 infection [31, 32]. Our findings, however, suggest better virus control and responsiveness to vaccination linked to the lower immunosuppressive potency of ciclosporin compared to tacrolimus. Although ciclosporin and tacrolimus suppress the immune system through the same main mechanism by preventing interleukin-2 production in T cells, ciclosporin and tacrolimus are chemically distinct molecules, and ciclosporin demonstrated weaker immunosuppressive potency compared to tacrolimus [33].

Our analysis further revealed CD19 + lymphocyte counts above 40/µL as a surrogate marker of positive immune responsiveness after two SARS-CoV-2 vaccination, but to a much lesser extent than MPA trough levels and calcineurin inhibitor use. While negatively correlated on the one hand with MPA trough levels, CD19^+^ lymphocytes and CD3^+^ lymphocytes positively correlate with eGFR reflecting reduced immune responsiveness with impaired kidney function as previously reported [34]. In line with our observation, CD19^+^ lymphocytes have previously been reported to be surrogate markers for immune competence [14].

The limitations of our study are the lack of systematic measurement of MPA-AUC, which has been reported to be the gold standard of TDM for MPA. We only had data on MPA-AUC by limited sampling strategy in a small subset of patients. Yet, correlations between MPA-AUC and trough levels have been shown in the literature and are in line with our observation in the limited cohort of patients. Moreover, trough level measurement is much more convenient in the daily routine and, therefore, easily implementable. We acknowledge that MPA dose reductions according to MPA trough levels need to be verified in a second step by using MPA-AUC measurement to ensure drug efficacy and limit the risk of rejections. Ideally, further studies testing the risk of rejections and development of *de novo* DSA following TDM-guided MPA dose reductions would help to support the safety of our recommendations. Even though our data suggest a cutoff of 2.5 mg/l as MPA through level discriminating vaccine responsiveness, we are aware of wide spreading of through levels in both groups underlining the need for larger studies to confirm our results. Yet, our data suggest that a trough level of >4 mg/L is a surrogate marker of drug overexposure, limiting the development of humoral vaccination responses. A further limitation related to the retrospective study designs is that MPA trough levels were measured when assessing SARS-CoV-2 vaccine responses and not at the time of vaccination. However, as the immunosuppressive regimen was not changed between the two time points, the results are expected to be the same. Additionally, we did not measure the neutralization capacity of anti-Sars-CoV-2 antibodies.

In conclusion, our study underlines the numerous and accumulating previous reports pointing towards an important role of MPA concerning reduced immune responsiveness in KTRs. Yet, it reaches beyond the correlation of MPA doses with immunoglobulin levels but suggests that individualizing MPA drug dosage to an MPA trough level below 2.5 mg/L might restore vaccine responsiveness to future vaccine doses. To ensure drug efficacy and prevent rejections, we recommend verification of dose reductions by MPA-AUC measurement in a second step. Prospective, randomized trials are needed to confirm our hypothesis and prove its safety concerning the risk of rejections and development of *de novo* DSA. Such confirmation of our observation in a larger population could have major implications not only for SARS-CoV-2 vaccination but for vaccinations in general—administered not only to KTRs but also for all other solid organ transplant recipients or patients under MPA therapy for autoimmune diseases.

## Data Availability

The data analyzed in this study is subject to the following licenses/restrictions: Data is available on request. Requests to access these datasets should be directed to thomas.schachtner@usz.ch.

## Abbreviations

AUC, Area under the curve; CI, Confidence interval; CNI, Calcineurin inhibitor; DSA, Donor specific antibody; EC-MPS, Enteric-coated mycophenolate sodium; eGFR, Estimated glomerular filtration rate; EHC, Enterohepatic cycling; HPLC, High pressure liquid chromatography; Ig, Immunoglobulin; IMPDH, inosine-5-monophosphate dehydrogenase; KTR, Kidney transplant recipient; MMF, Mycophenolate mofetil; MPA, Mycophenolic acid; NP, Nucleoprotein; mTOR, Mammalian target of rapamycin; OR, Odds ratio; ROC, receiver operating characteristic; SARS-CoV-2, Severe acute respiratory syndrome coronavirus-2; TDM, Therapeutic drug monitoring.
