# Supplementary material for: Therapeutic Drug Monitoring of Mycophenolic Acid Identifies Kidney Transplant Recipients Responsive to Two SARS-CoV-2 mRNA Vaccine Doses
Source: Transpl Int. 2023 Jun 28;36:11286. doi: 10.3389/ti.2023.11286 (PMC10336200; doi:10.3389/ti.2023.11286)
Supplement: Supplementary file 1 [file DataSheet1.docx]

**SUPPLEMENT**

**TABLES**

**Supplement Table 1:** Impact of collinearity of the independent variables associated with seroconversion

|  | **Collinearity statistics** | | |
| --- | --- | --- | --- |
|  | **Tolerance** | **VIF** |  |
| eGFR at the time of  1^st^ vaccination, ml/min/1.73m^2^ | 0.806 | 1.241 |  |
| Mycophenolic acid trough level <2.5mg/l | 0.747 | 1.339 |  |
| Total lymphocytes, /µl | **0.234** | **4.274** |  |
| CD3+ lymphocytes, /µl | **0.244** | **4.105** |  |
| CD19+ lymphocytes, /µl | 0.682 | 1.465 |  |
| Ciclosporin as calcineurin inhibitor | 0.920 | 1.087 |  |

**SUPPLEMENT FIGURES**

**Supplement Figure 1**


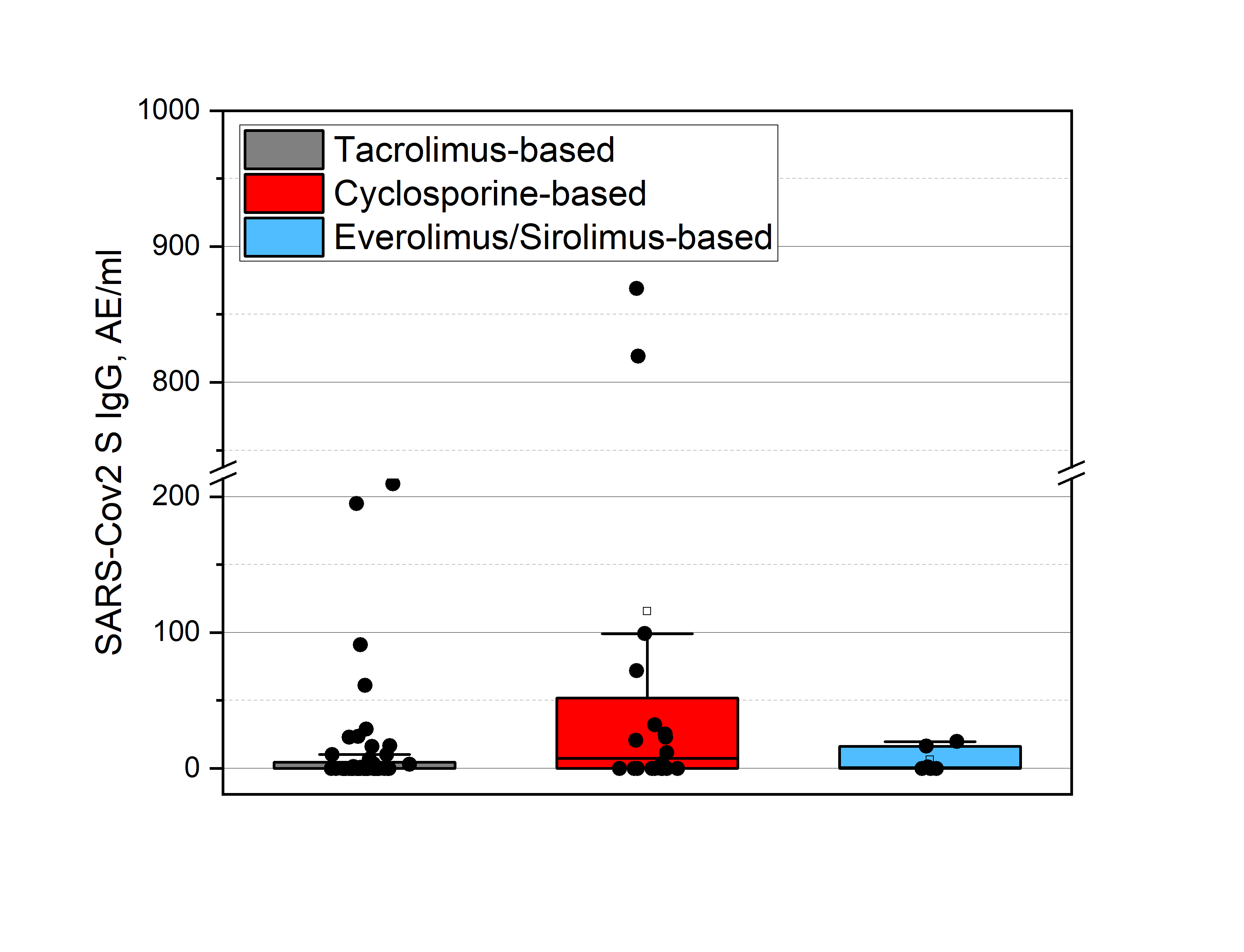


**Supplement Figure 2A**


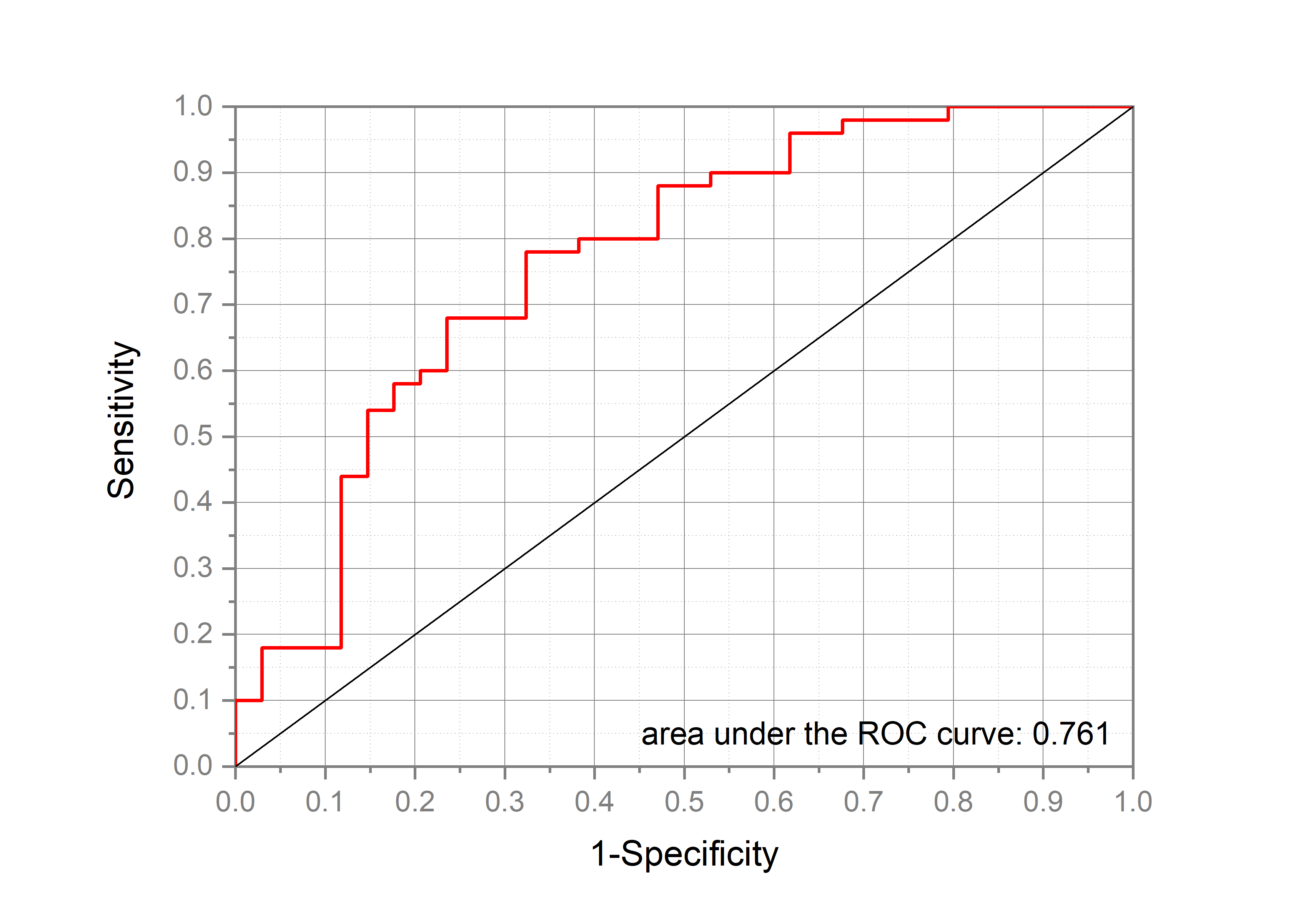


**Supplement Figure 2B**


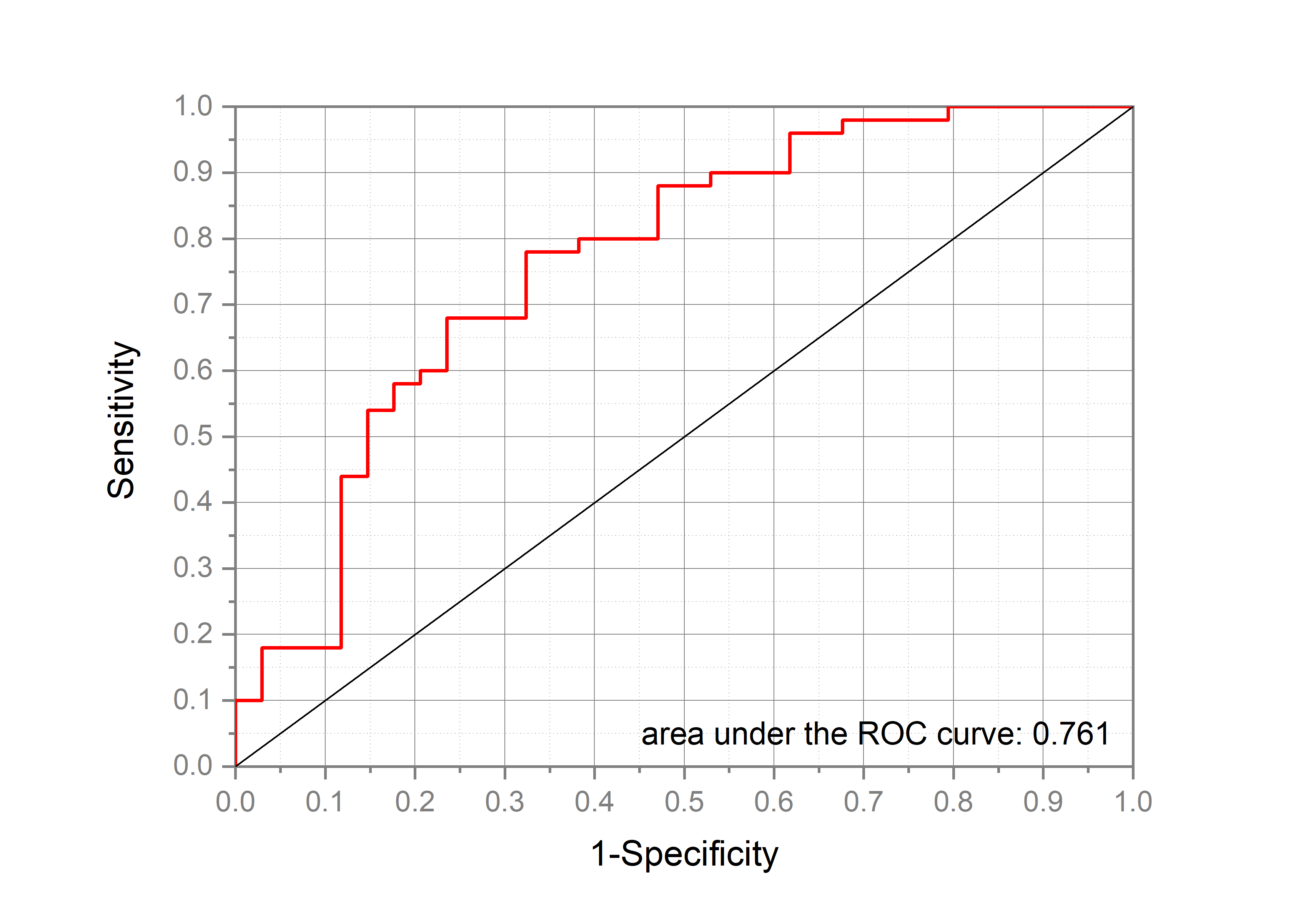


**Supplement Figure 2C**


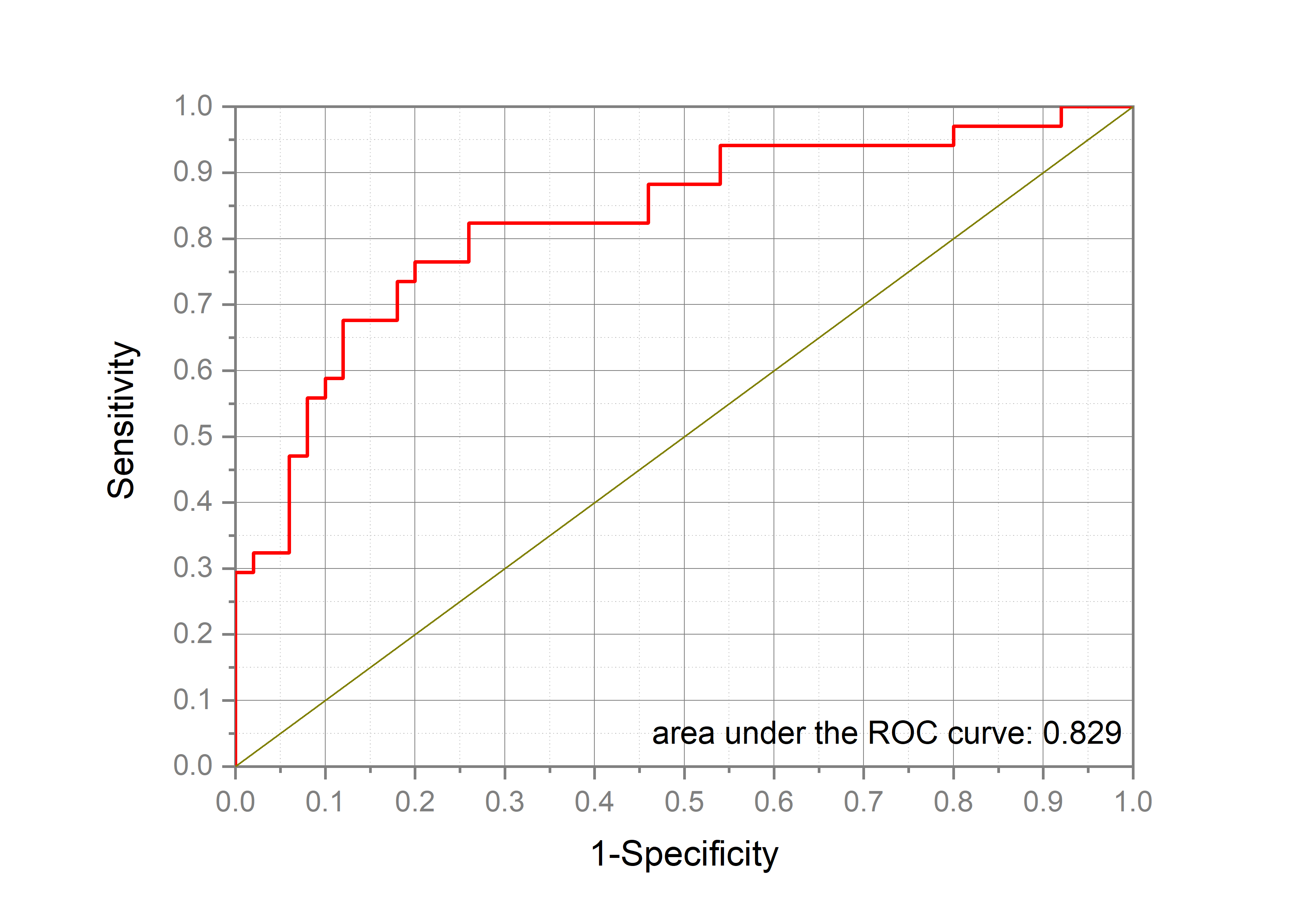


**Supplement Figure 3**


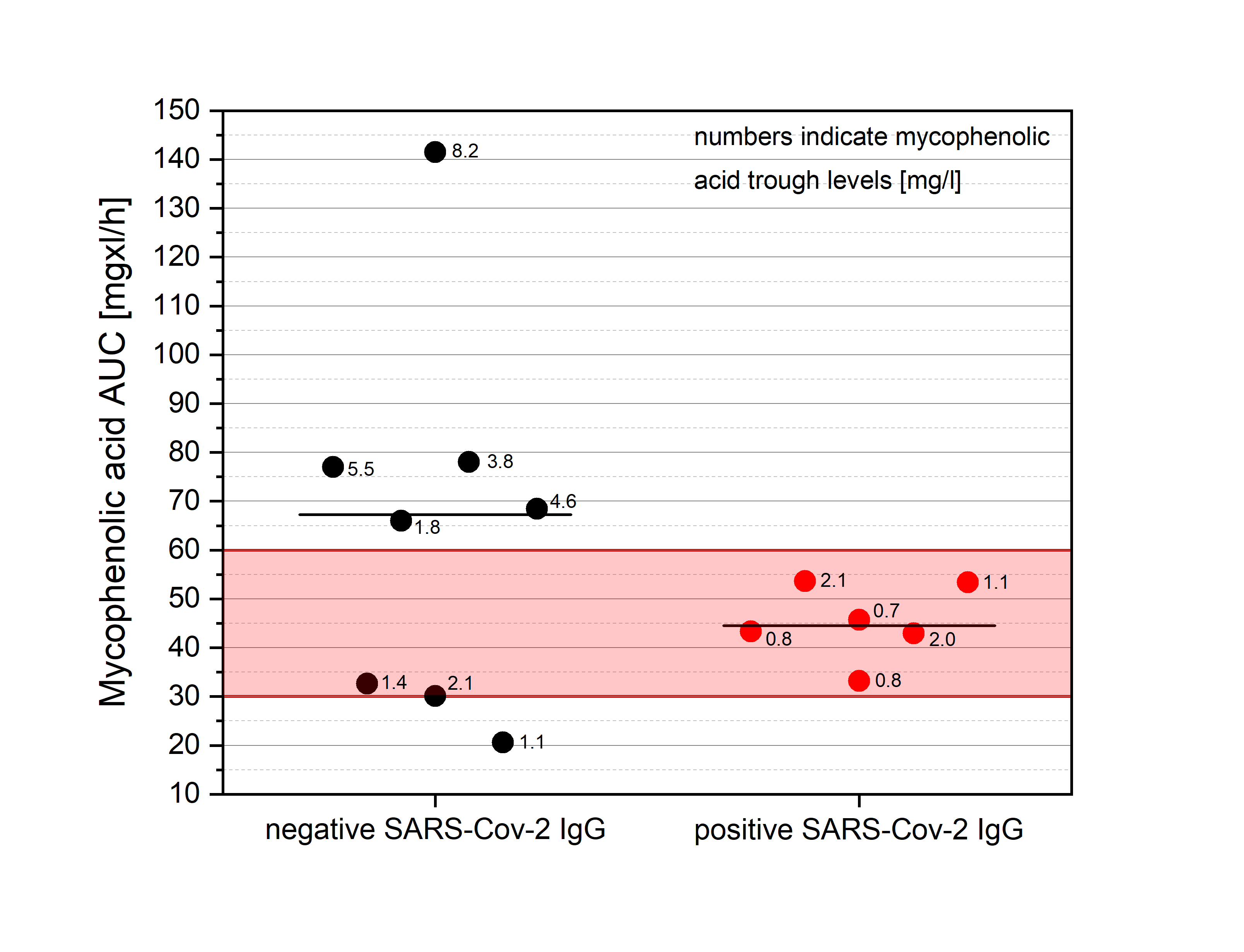


**Supplement Figure 4A**


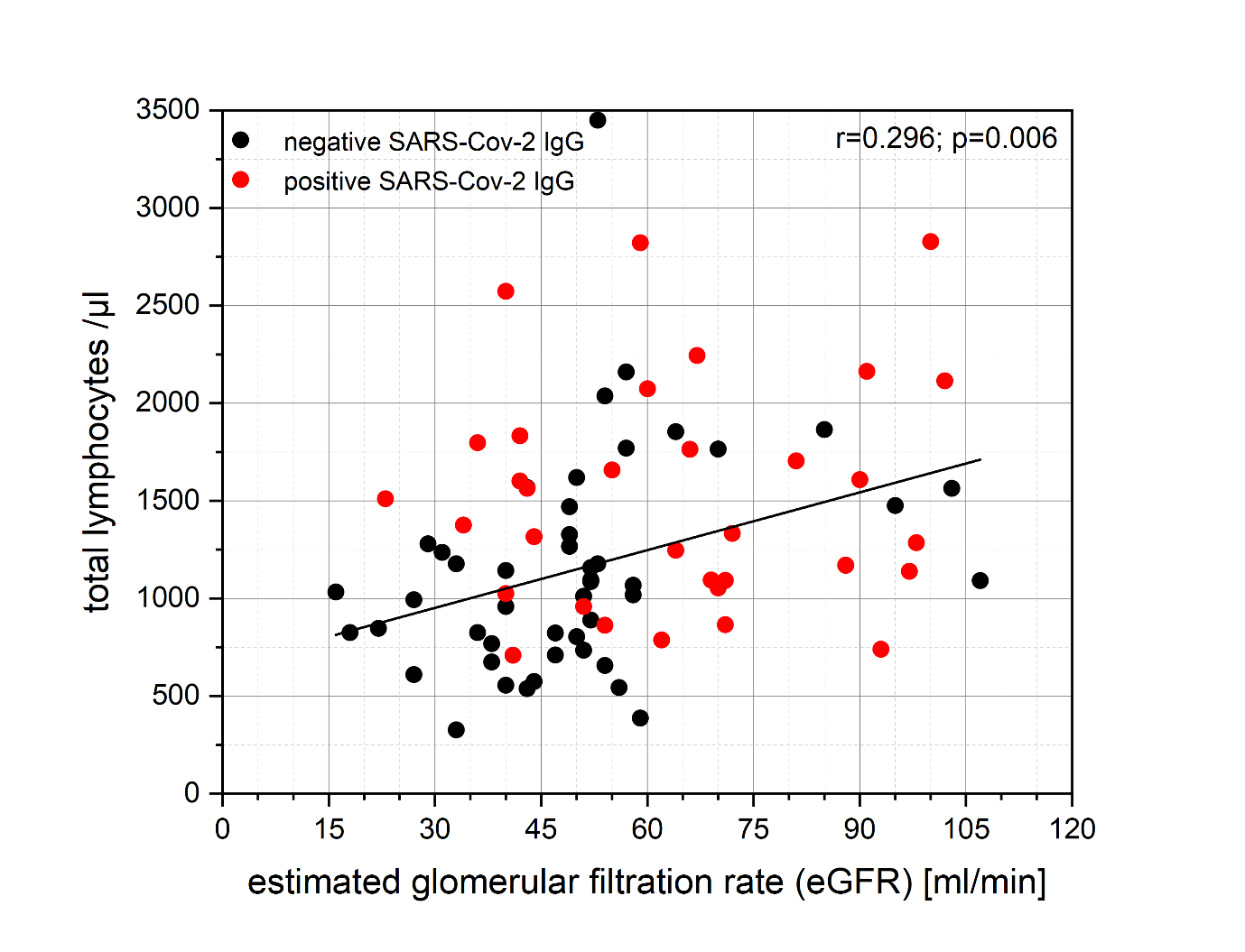


**Supplement Figure 4B**


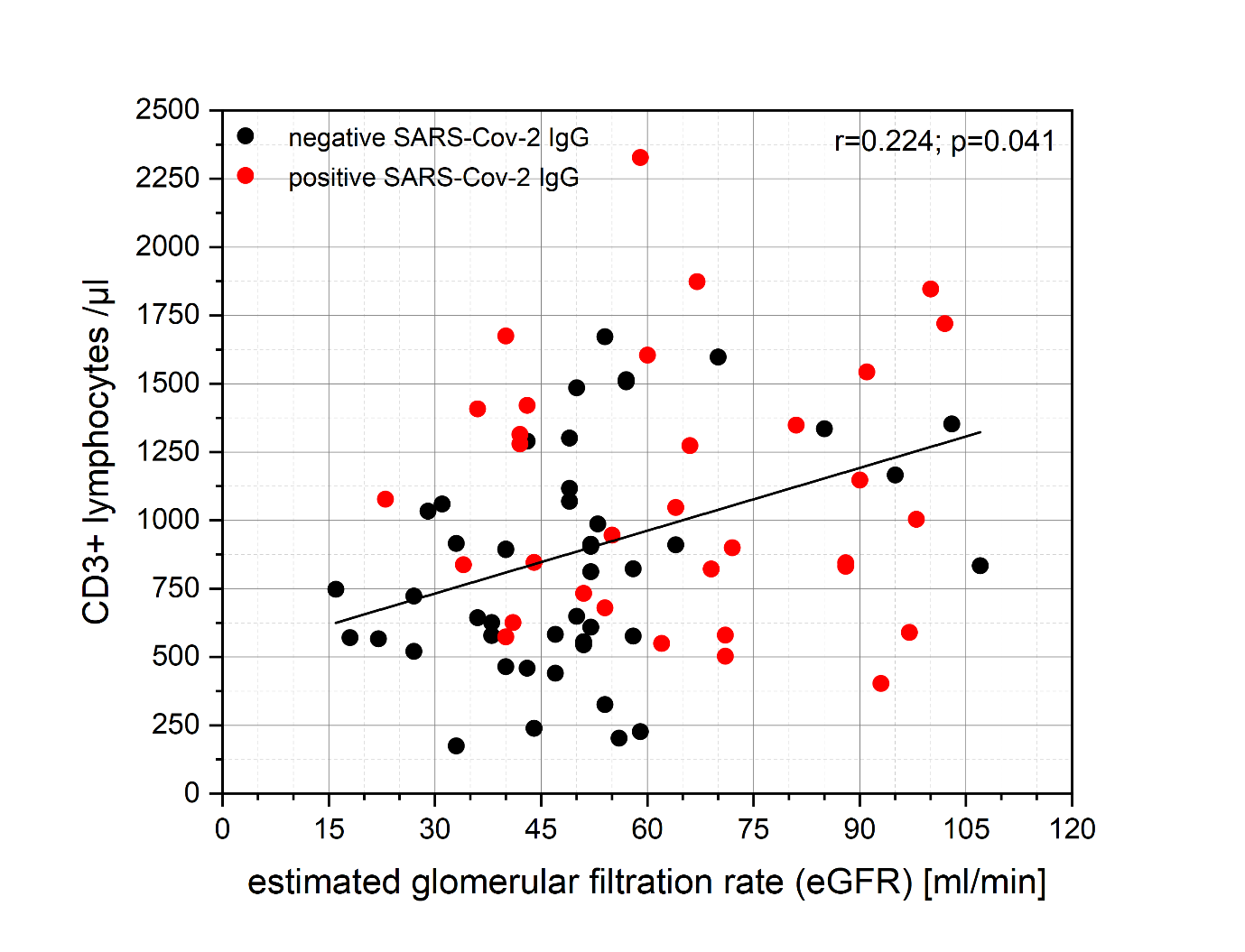


**Supplement Figure 4C**


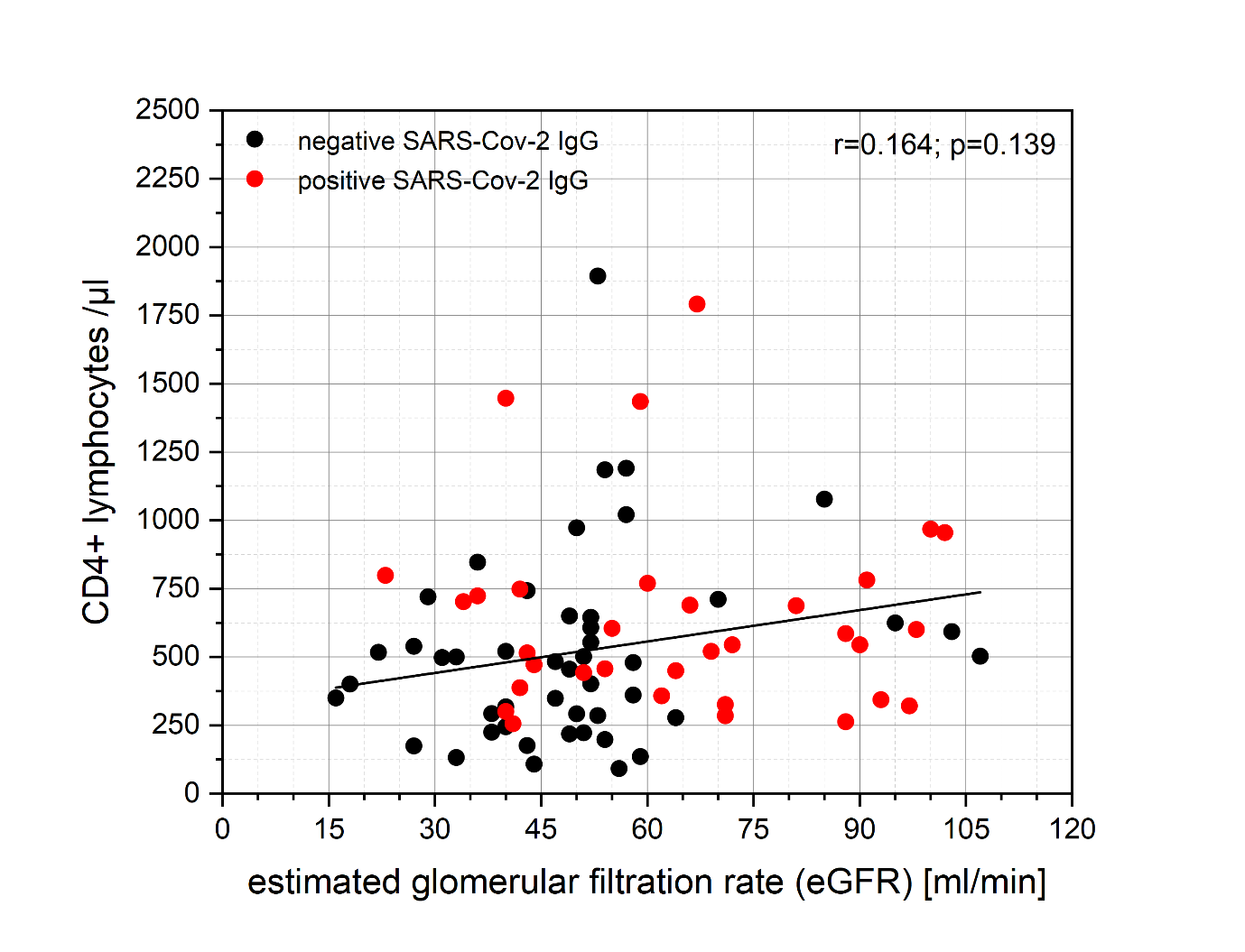


**Supplement Figure 4D**


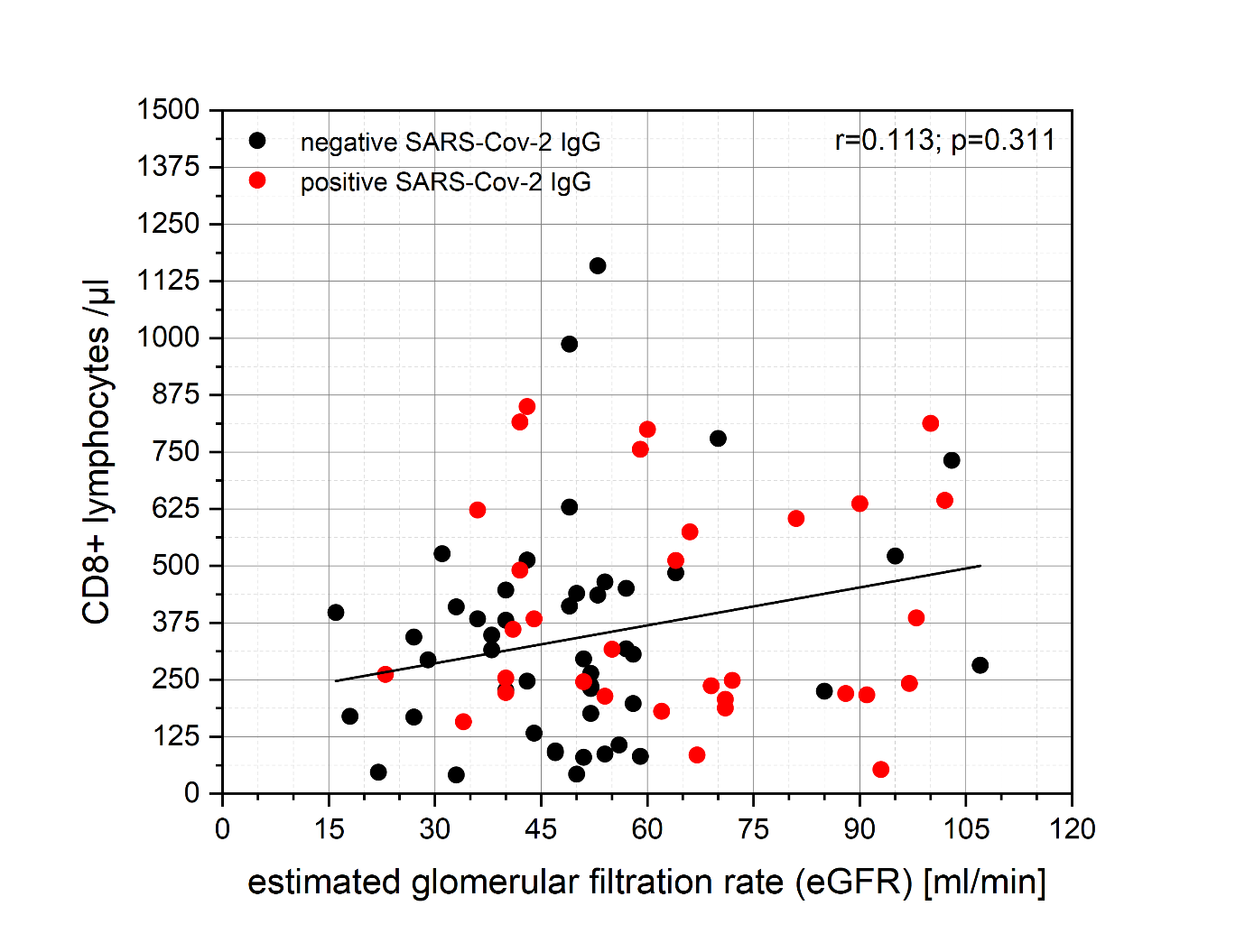


**Supplement Figure 4E**


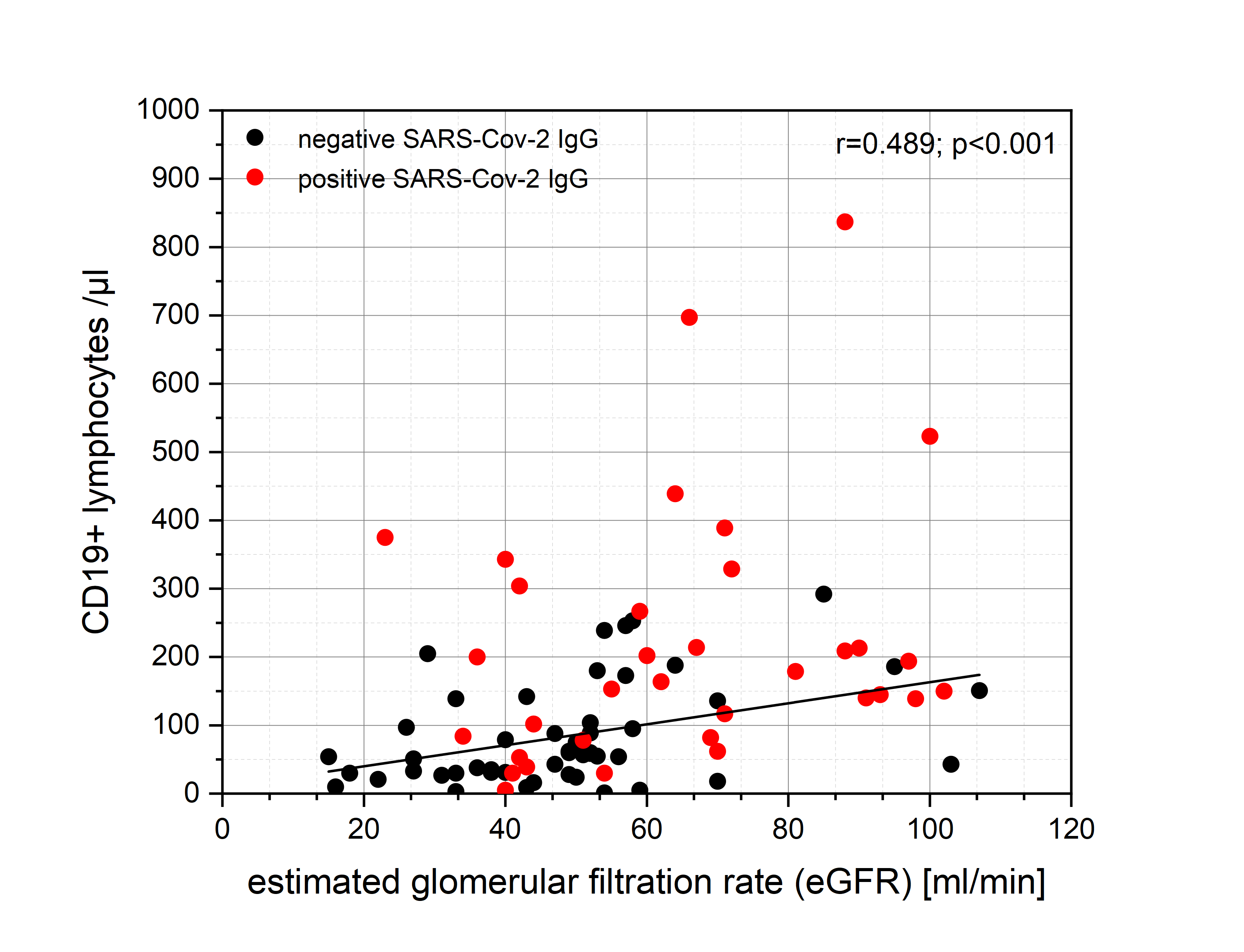


**SUPPLEMENT FIGURE LEGENDS**

**Supplement Figure 1. SARS-Cov-2 IgG in relation to type of calcineurin inhibitor or mTOR inhibitor use.**

**Supplement Figure 2A-C. ROC curve analysis for (A) MPA trough levels, (B) CD19+ lymphocytes, and (C) the combination of MPA trough levels and CD19+ lymphocytes.** 2A MPA trough levels of less than 2.5mg/l identify KTRs with positive SARS-Cov-2 IgG after two doses of SARS-Cov-2 mRNA vaccine with a sensitivity and specificity of 78.0% and 67.6%, respectively (area under the ROC curve: 0.761). 2B CD19+ lymphocytes of greater than 100/µl identify KTRs with positive SARS-Cov-2 IgG after two doses of SARS-Cov-2 mRNA vaccine with a sensitivity and specificity of 72.7% and 70.6%, respectively (area under the ROC curve: 0.755). 2C MPA trough levels of less than 2.5mg/l together with CD19+ lymphocytes of greater than 40/µl identify KTRs with positive SARS-Cov-2 IgG after two doses of SARS-Cov-2 mRNA vaccine with a sensitivity, specificity, positive predictive value, and negative predictive value of 67.6%, 88.0%, 82.1%, and 80.4% respectively (area under the ROC curve: 0.829).

**Supplement Figure 3. Distribution of MPA-AUC in KTRs with positive/negative SARS-Cov-2 IgG.** MPA trough levels are shown for each MPA-AUC (13 patients: MMF+Tacrolimus; 1 patient EC-MPA+Tacrolimus). The red shading shows the MPA target range of 30-60mgxh/L.

**Supplement Figure 4A-E. Correlation of eGFR and (A) total lymphocytes, (B) CD3+ lymphocytes, (C) CD4+ lymphocytes, (D) CD8+ lymphocytes, and (E) CD19+ lymphocytes.**
